# Co-Operation

**Published:** 1914-03-21

**Authors:** 


### CO-OPERATION.

The doctor's wife is to be found in all communities, and collectively they must have a wide knowledge of co-operative methods, including the great co-operative societies in the North, which have proved such a blessing to the heads of humbler households. We expect, too, many are familiar with the Mammoth Stores Organisations to be found in the greater centres throughout the country, and especially in London One of the chief factors in success in life is co-operation. This principle has long been the keynote of many of the industrial enterprises organised by men. It is only in recent years that co-operation has been resorted to by women even in the smallest degree. If, however, the doctor's wife will courageously and firmly grip this principle, she should speedily realise in practice that it is the fundamental basis on which increased life, health, means, and prosperity can be built up. We are conscious that the problem of co-operation among women in their home life can be made one of the most successful of enterprises. But to succeed women must awaken and join hands. This problem has not hitherto had adequate thought brought to bear upon it, and we are confident that, if the doctors' wives are ready, co-operation and the increase of her resources can be made worthily to fill a woman's life and add materially to her happiness and usefulness as a mother and a wife.

**It is with this conviction and for these reasons that we venture to invite those heads of doctors' households into whose hands this Section may fall to fill in the coupon and return it to the office in due course.**

